# Field Epidemiology Training Programmes in Africa - Where are the Graduates?

**DOI:** 10.1186/1478-4491-8-18

**Published:** 2010-08-09

**Authors:** David Mukanga, Olivia Namusisi, Sheba N Gitta, George Pariyo, Mufuta Tshimanga, Angela Weaver, Murray Trostle

**Affiliations:** 1 African Field Epidemiology Network, P. O. Box 12874 Kampala, Uganda; 2 Global Health Workforce Alliance, World Health Organisation, Avenue Appia 20, CH-1211 Geneva 27, Switzerland; 3 Department of Community Medicine, University of Zimbabwe, P.O. Box MP167, Mount Pleasant, Zimbabwe; 4 Public Health Institute, United States Agency for International Development Global Health Fellows Program, Washington DC, USA; 5 United States Agency for International Development, Washington DC, USA

## Abstract

**Background:**

The current shortage of human resources for health threatens the attainment of the Millennium Development Goals. There is currently limited published evidence of health-related training programmes in Africa that have produced graduates, who remain and work in their countries after graduation. However, anecdotal evidence suggests that the majority of graduates of field epidemiology training programmes (FETPs) in Africa stay on to work in their home countries--many as valuable resources to overstretched health systems.

**Methods:**

Alumni data from African FETPs were reviewed in order to establish graduate retention. Retention was defined as a graduate staying and working in their home country for at least 3 years after graduation. African FETPs are located in Burkina Faso, Ethiopia, Ghana, Kenya, Nigeria, Rwanda, South Africa, the United Republic of Tanzania, Uganda and Zimbabwe. However, this paper only includes the Uganda and Zimbabwe FETPs, as all the others are recent programmes.

**Results:**

This review shows that enrolment increased over the years, and that there is high graduate retention, with 85.1% (223/261) of graduates working within country of training; most working with Ministries of Health (46.2%; 105/261) and non-governmental organizations (17.5%; 40/261). Retention of graduates with a medical undergraduate degree was higher (Zimbabwe 80% [36/83]; Uganda 90.6% [125/178]) than for those with other undergraduate qualifications (Zimbabwe 71.1% [27/83]; Uganda 87.5% [35/178]).

**Conclusions:**

African FETPs have unique features which may explain their high retention of graduates. These include: programme ownership by ministries of health and local universities; well defined career paths; competence-based training coupled with a focus on field practice during training; awarding degrees upon completion; extensive training and research opportunities made available to graduates; and the social capital acquired during training.

## Background

A key ingredient to achieving improved health outcomes is stronger health systems, including an adequate health workforce [1,2]. There is evidence of a direct and positive causal link between numbers of health workers and health outcomes [3,4]. The World Development Report 2004 [5] states that without improvements to the health workforce, the health-related Millennium Development Goals cannot be achieved. In many countries, the effects of insufficient development of the health workforce are aggravated by migration and a mounting burden of disease [5]. The current shortage of health workers, particularly in sub-Saharan African countries, threatens the realization of plans for scaling up interventions to control the spread of diseases such as HIV/AIDS, malaria, and tuberculosis [6].

Available data from cohorts of graduates of medical and other allied health science schools in Africa show that at least 40% of graduates move on to work outside their home countries [7,8]. There is little or no evidence of medical or related training programmes that have been able to produce graduates, the majority of whom stay on to work in their home countries in Africa, or in developing countries. Such programmes could provide valuable lessons and potential solutions to the problem of massive brain drain of the health workforce in Africa and other developing regions of the world. On the other hand, anecdotal evidence suggests that the majority of graduates of field epidemiology training programmes (FETPs) in Africa stay on to work in their home countries. We reviewed alumni data from African FETPs in order to establish their graduate retention in the wake of acute health worker shortages.

### Field epidemiology training programmes in Africa

FETPs help countries develop and implement dynamic cost-effective public health strategies to improve and strengthen their public health systems and infrastructure [2]. These training programmes offer competency-based training, comprising field epidemiology, health services management, disease control, health communication, and prevention effectiveness.

The first FETP in Africa was established in Zimbabwe in 1993, followed by Uganda in 1994. These programmes were established as partnerships between the respective Ministries of Health (MoH), universities and district local governments, with financial support from the Rockefeller Foundation. They came to be known as 'public health schools without walls' [9]. Programmes shared experiences, training curricula and materials, staff, and undertook joint field epidemiology projects [10,11].

Trainees spend 25-30% of the 2-year long programme mastering content through didactic classes. The underlying theme of the FETP model is that trainees 'learn by doing', and therefore the remainder of the time is spent gaining hands-on experience through a field placement. This is usually in a MoH service department or unit, located either centrally (e.g., the disease surveillance department, immunization program, or the HIV program) or in the health departments in the provinces or districts. There, trainees (or residents) are closely supervised with emphasis on acquisition of skills and competencies.

Field epidemiology and laboratory training programmes (FELTPs) add a laboratory component; training field epidemiologists and public health laboratory scientists jointly to address public health problems. In 2004, the Kenya FELTP was established with financial support from the Ellison Medical Foundation provided through the CDC Foundation, as a partnership with the Kenya Ministry of Health, and with a regional mandate that included training Ghanaian, Southern Sudanese, Tanzanian and Ugandan health professionals. In 2007, the South Africa FELTP was started as a partnership between the South African government's National Department of Health, the National Institute for Communicable Diseases of the National Health Laboratory Service, the University of Pretoria, and CDC's Global AIDS Program (GAP), with funding from the President's Plan for Emergency AIDS Relief (PEPFAR).

In 2008 the Tanzanian and Nigerian FELTPs were established. The United Republic of Tanzania FELTP is a partnership between the Ministry of Health and Social Welfare of the United Republic of Tanzania, the Muhimbili University College of Health and Allied Sciences, the United States Agency for International Development (USAID), CDC, PEPFAR, and the African Field Epidemiology Network (AFENET)--which is a networking and service alliance of African FETPs and FELTPs, and several other local and international partners. The Nigeria FELTP is a partnership between the Federal Ministry of Health of Nigeria, the Federal Ministry of Agriculture and Water Resources, the University of Ibadan, Ahmadu Bello University, USAID, CDC, and AFENET. The Nigeria FELTP is the first program to have joint training for field epidemiologists, public health laboratory scientists, and veterinary field epidemiologists (online at http://www.nigeria-feltp.net).

In 2009, a new FETP was established in Ethiopia. This year (2010), the Rwanda FELTP and the West Africa FELTP based in Ouagadougou were established. The West Africa Programme, comprising of Burkina Faso, Mali and Togo is the first Francophone FELTP in Africa.

The success and achievements of FETPs and FELTPs has attracted trainees from other countries in Africa, and also the United Kingdom, U.S.A., Oceania, and Japan, as well as having precipitated demand for field epidemiologists, public health laboratory scientists, and public health specialists trained through this model. This demand has led to a desire by many African countries to start their own FETPs or FELTPs. Angola, Cameroon, Central Africa Republic, the Democratic Republic of the Congo, and Mozambique have expressed interest in beginning their own programmes. Assessments to develop programmes in these countries were recently completed.

### What do FETP graduates do?

Graduates play a central role in public health surveillance, disease control and in the design, implementation, and evaluation of various public health programmes (e.g., in malaria, tuberculosis, and HIV/AIDS, maternal and child health and immunisation programs) and in outbreak investigation and control. FETP alumni have risen to top leadership positions in ministries of health, non-governmental organizations, and other health agencies. They also have implemented cross-border public health surveillance systems that have contributed significantly to reducing transmission of diseases and promoted enforcement of the International Health Regulations.

Most district and provincial medical officers in Zimbabwe and Uganda are FETP graduates. They are responsible for the planning and delivery of routine health services in their jurisdictions. Many of the disease control programmes in countries with FETPs or FELTPs are managed by graduates. The graduates have had a great impact in the implementation and maintenance of disease surveillance systems.

When the World Health Organisation (WHO) launched the Integrated Disease Surveillance and Response (IDSR) strategy in 1998 in the African region, FETP graduates were subsequently recruited into key positions and were instrumental to the success of IDSR in the FETP-host countries in Africa. Disease surveillance, outbreak investigation and management, and production and circulation of IDSR bulletins in Zimbabwe, Uganda, Kenya, the United Republic of Tanzania, and Ghana is the function of FETP graduates working within the Epidemiology Units of the ministries of health.

Disease epidemics continue to ravage sub-Saharan Africa. Graduates have played a key role in the investigation and response to epidemics in their countries. Selected examples include: an Ebola outbreak in Uganda [10] in 1998; an aflatoxin poisoning outbreak in Kenya [11] in 2004; Rift Valley Fever outbreaks in Kenya [12] and the United Republic of Tanzania in 2007; and cholera in Zimbabwe in 2009.

## Methods

Each FETP maintains a database of its graduates and trainees. In addition, AFENET maintains an aggregate database of all alumni and current trainees from member programmes. All programme databases use MS Excel or Epi Track, which is an MS Access-based management information tool that has been provided to FETPs and FELTPs by CDC to aid the evaluation of program impact on public health systems, and ultimately on the health of the public [13]. Programme administrative assistants maintain and regularly update the databases (at least once a year).

From admission of FETP trainees, through their progress during the programme and into the post-graduation period, data is captured annually via email or telephone.

The programme administrative assistants abstracted data for the period 1993 to 2004 on the following variables: name, gender, year of enrollment, year of graduation, current workplace and designation, current location/country, background training (degree/diploma attained). They then sent it to us as MS Excel documents. Data from the different programmes were aggregated into one MS Excel file. In order to measure the extent of retention among FETP alumni, we calculated the proportion of graduates that were currently working within their home country. Retention was defined as a graduate staying and working in their home country for at least 3 years after graduation. Data were analysed by programme and year of enrollment on the various study variables in MS Excel. Percentages were computed for the different study variables and are presented in the next section as text, tables and charts.

The Kenya, Nigeria, South Africa, and the United Republic of Tanzania FELTPs were excluded, as none of them had produced graduates for more than the 3-year cutoff at the time of our analysis.

## Results

### FETP enrolment (by number and undergraduate qualification of trainees)

**Figure 1:**
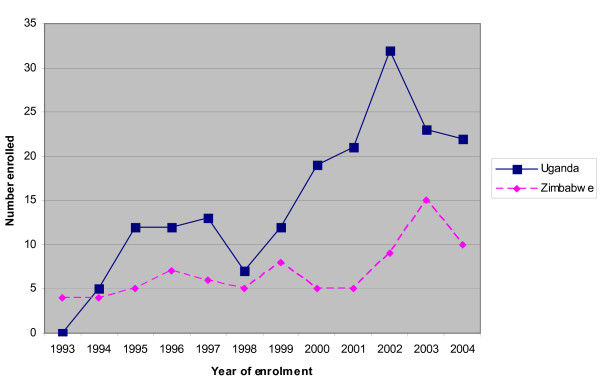
**Annual trainee enrolment by programme (1993-2004)**.

The total number of graduates from the Uganda and Zimbabwe programmes between 1993 and 2004 is 261 (Zimbabwe 83, Uganda 178). Zimbabwe's first cohort (1993) had a total of four trainees, while Uganda's (1994) had five trainees. Trainee enrolment has increased over the years as shown in Figure 1.

A total of 83 trainees were enrolled into the Zimbabwe programme between 1993 and 2004, while 178 were enrolled into the Uganda programme between 1994 and 2004.

The distribution of enrolment by undergraduate training is shown in Table 1. The majority of trainees in both programs were medical doctors.

**Table 1:** Undergraduate qualifications of trainees enrolled into the Zimbabwe and Uganda FETPs, 1993-2004

	Zimbabwe		Uganda	
**Undergraduate qualification**	**Frequency (N = 83)**	**Percentage**	**Frequency (N = 178)**	**Percentage**

MD	45	54.2	138	77.5
BSc (Bachelor of Science)	36	43.4	14	7.9
BVM (Bachelor of Veterinary Medicine)	02	2.4	2	1.1
BDS (Bachelor of Dental Surgery)	00	0.0	11	6.2
Social sciences	00	0.0	13	7.3

### Retention within home country after training

**Figure 2:**
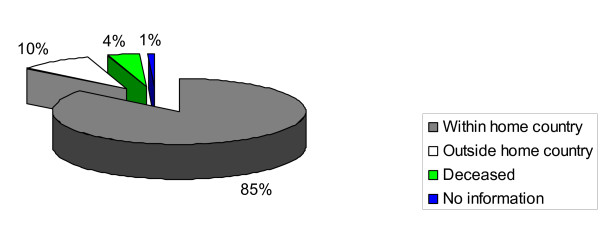
**Current locations of Uganda and Zimbabwe FETP graduates (1993-2004)**.

Of all FETP graduates, 85% are working within their home country as shown in Figure 2.

A review of retention for the initial five cohorts for each of the two programmes showed that for Zimbabwe (1993-1997 enrolments), retention within country was (42%, 11/26), working abroad (42%, 11/26), and deceased (15%, 4/26). For Uganda (1994-1998 enrolments), retention within country was (86%, 42/49), working abroad (7%, 2/29), deceased (6%, 3/49), and those with no information (4%, 2/49).

### Graduate retention by cohort

We assessed alumni retention within country by class cohort. The retention of graduates varied among the different programmes as shown in Table 2.

**Table 2:** Proportion of graduates working within home country by class cohort

Cohort (Year)	Proportion within home country Frequency (%)	
	**Uganda**	**Zimbabwe**

1993-1995	0 (0)	1 (25%)
1994-1996	5 (100%)	3 (75%)
1995-1997	11 (91.7%)	1 (20%)
1996-1998	12 (100%)	5 (71.4%)
1997- 1999	10 (76.9%)	1 (20%)
1998-2000	4 (57.1%)	3 (60%)
1999-2001	10 (83.3%)	6 (75%)
2000-2002	14 (73.7%)	4 (80%)
2001-2003	20 (95.2%)	5 (100%)
2002-2004	30 (93.8%)	9 (100%)
2003-2005	23 (100%)	15 (100%)
2004-2006	21 (95.5%)	10 (100%)

### Retention of FETP graduates by undergraduate qualification/training

Table 3 shows the retention of graduates by undergraduate qualification. The majority of graduates with a medical undergraduate degree from both Zimbabwe (80%) and Uganda (90.6%) were working within their home country at the time of this review. For graduates with an undergraduate degree other than medicine, the retention was lower: Zimbabwe = 71.1%; Uganda = 87.5%.

**Table 3:** Graduate retention by undergraduate qualification, Zimbabwe and Uganda FETPs, 1993-2004

		Zimbabwe		Uganda	
	Location of graduates	Frequency (N = 83)	%	Frequency (N = 178)	%
Undergraduate training/qualification					
MD	Within home country	36	80.0	125	90.6
	Outside home country	6	13.3	7	5.1
	Deceased	3	6.7	4	2.9
	No information	0	0	2	1.4
**Total - MD**		45	100.0	138	100.0

Other qualifications	Within home country	27	71.1	35	87.5
	Outside home country	10	26.3	2	5.0
	Deceased	1	2.6	3	7.5
	No information	0	0	0	0
**Total - OTHER**		38	100.0	40	100.0

### Sectors where graduates are employed

Out of 261 graduates from the Uganda and Zimbabwe programmes, 223 (85%) are employed within their home countries. These graduates are working for a number of sectors and organisations: ministries of health (105, 47.1%); non-governmental organisations (40, 17.9%); universities (25, 11.2%); international agencies (24, 10.8); local governments (14, 6.3%); other government ministries like agriculture, finance; internal affairs and defence (7, 3.1%); and the private sector (8, 3.6%).

## Discussion

This analysis shows that the majority (85%) of graduates from 2 FETPs in Africa have been retained by their countries. This is in agreement with anecdotal evidence that suggests that the majority of graduates of FETPs stay on to work in their countries, but is in contrast to earlier studies that showed close to 40% of medical graduates from Africa were living abroad [7]. For example, more than 80% of the Uganda FETP alumni that graduated in 1997 are still working in Uganda today, 10 years after graduation, as compared to only 60% of the medical school graduates that were still in Nigeria [8]. Even the Zimbabwe programme has over the years registered a healthy retention of its graduates in the country despite the worsening economic situation.

One of the requirements for admission into the programmes is at least 2 years' field experience after the first degree. Trainees join the programmes having established a career and a social network within their country. These conditions are thought to play a major role in the graduates not moving abroad, as that would be disruptive to their careers, family and social networks.

This review provides at least four possible explanations for health worker retention that may be applicable to other human resources for health training programmes in Africa and other developing countries:

a) The first is on programme ownership. Often, human resources rank low on the agenda of both governments, bilateral and multilateral agencies. Although difficulties with workforces frustrate most social sectors; health workers have been particularly neglected. The workforce in many low income countries is adversely affected by severe under-investment from the national funds as well as from external resources [14]. All the African FETPs and FELTPs are co-owned by the MoH, a local university and other stakeholders. The MoH contributes to the training (e.g., in terms of availing training sites, tuition fees, mentors for the trainees as well as other resources). FETP co-ownership by the MoH has ensured that training remains relevant to the needs of the ministry and the country's health sector; hence graduates get placements easily within the country of training.

b) The second is on the importance of having a well defined career path. In order to reduce migration of health care workers from developing countries to developed nations, we must address the issues that make developed countries attractive. One of the most frequently cited reasons for seeking employment abroad is a desire for postgraduate training and career development [15]. In the formative stages of the FETPs, the respective university, MoH and other stakeholders hold meetings that define the career paths of the programme graduates. Consequently, upon graduation, positions are available within the MoH structure and career progression is well defined. This is probably one of the major contributing factors to graduate retention. The higher retention of medical doctors compared to other cadres may partly be explained by clearer and more attractive career paths in public health for the former.

c) The third is the field-based training model adopted by FETPs. FETPs focus on competency-based training and field training, with trainees spending 70-75% of their time at a field site, which may be a district or provincial health office, or a disease control program within the MoH. This acclimatises trainees to the real world and working conditions, helping them realize that they can develop a viable career within this kind of environment.

d) Finally, the FETP model offers trainees social capital [16] and innovative incentives. Trainees have opportunities to rotate through the MoH and, in the case of Zimbabwe, trainees have monthly meetings where they make presentations to MoH officials, sharing their work experiences and challenges. These interactions with senior MoH officials, as well as MoH development partners, provide trainees with invaluable future professional contacts and potential employers. This social capital has been a major determinant of graduate employment and consequently, retention. Closely related to this are the teaching and research opportunities availed to graduates as part-time lecturers or research fellows in training institutions when they complete their own training.

## Conclusions

This report has described how African FETPs have shown that when you recruit trainees locally, recruit trainees with field experience, train them in a competency-based training program locally, and deploy during the training locally, then the likelihood that they will stay in the country after graduation is greatly enhanced. This is in contrast to training people abroad--within health systems that are different from the health systems they will eventually have to work in upon graduation-- and then having to re-train them on the local health systems if they return to the country of origin. Anecdotal evidence suggests that those who go back are often frustrated by their inability to work in health systems that they did not train in and eventually join the brain drain.

We therefore recommend that countries, governments and training institutions consider adopting this approach to capacity development.

### Future research

It would be important for future studies to examine how competencies acquired during training meet the needs of the graduates' current jobs and therefore identify which gaps need to be addressed. There is also need to examine career and professional development of FETP graduates such as publications and job promotions since graduation.

## Conflict of interests

The authors declare that they have no competing interests.

## Authors' contributions

DM: Contributed to study conception and design, acquisition and interpretation of data, revised the article for intellectual content, and approved the article to be published.

ON: Contributed towards study conception, acquisition of data, analysis and interpretation of data, drafting the article and approval of the version to be published.

SG: Contributed towards study design, analysis and interpretation of data, drafting the article and revising it for important intellectual content, and final approval of the version to be published.

GP: Contributed towards the conception and design of this study, reviewed the article for important intellectual content, and approval of the version to be published.

MT: Contributed towards the conception and design of this study, reviewed the article for important intellectual content, and approval of the version to be published.

AW: Contributed towards the conception of this study, reviewed the article for important intellectual content, and approval of the version to be published.

MT: Contributed towards the conception of this study, reviewed the article for important intellectual content, and approval of the version to be published.

## References

[B1] Travis P Bennett S Haines A Pang T Bhutta Z Hyder AA Pielemeier NR Mills A Evans T Overcoming health- systems constraints to achieve the Millennium Development Goals The Lancet 2004 364 9437 900 906 10.1016/S0140-6736(04)16987-0 15351199

[B2] Nsubuga P White M Fontaine R Simone P Training programmes for field epidemiology The Lancet 2008 371 9613 630 631 10.1016/S0140-6736(08)60281-0 18295009

[B3] Hongoro C McPake B How to bridge the gap in human resources for health The Lancet 2004 364 9443 1451 1456 10.1016/S0140-6736(04)17229-2 15488222

[B4] Anand S Bärnighausen T Human resources and health outcomes: cross-country econometric study The Lancet 2004 364 9445 1603 1609 10.1016/S0140-6736(04)17313-3 15519630

[B5] World Bank World Development Report 2004: Making Services Work for Poor People 2003 Washington, DC: World Bank

[B6] Inke M Ingo I Health Worker Motivation in Africa: the role of non-financial incentives and human resource management tools Human Resources for Health 2006 4 1 24 10.1186/1478-4491-4-24 PMC1592506 16939644

[B7] Dambisya Y The fate and career destinations of doctors who qualified at Uganda's Makerere Medical School in 1984: retrospective cohort study BMJ 2004 329 600 601 10.1136/bmj.38134.524387.AE PMC516657 15297304

[B8] Chikwe I Ike A Enyinnaya A Nigerian medical graduates: where are they now? The Lancet 2005 365 9474 1847 1848 10.1016/S0140-6736(05)66612-3 15924978

[B9] Tulane University Public Health Schools Without Walls http://www.tulane.edu/~phswow/(accessed on October 19, 2007)

[B10] Oyok T Odonga C Mulwani E Abur J Kaducu F Akech M Outbreak of Ebola Haemorrhagic Fever- Uganda, August 2000-January 2001 Morbidity and Mortality Weekly Report 2001 50 05 73 7 11686289

[B11] Nyikal J Misore A Nzioka C Njuguna C Muchiri E Njau J Outbreak of Aflatoxin Poisoning in Eastern and Central Provinces, Kenya, January -July 2004 Morbidity and Mortality Weekly Report 2000 53 34 790 793 15343146

[B12] Centres for Disease Control and Prevention (CDC) Rift Valley fever outbreak-Kenya, November 2006-January 2007 Morbidity and Mortality Weekly Report 2007 56 4 73 6 17268404

[B13] Elbon SM Jones DS Nsubuga P EpiTrack AFENET. [Computer software] (2009) Atlanta, GA: Centers for Disease Control

[B14] Vasant N Hilary B Pablos-Mendez Ariel Adams Orvill Dussault Gilles Elzinga Gijs Responding to the global human resources crisis The Lancet 2004 363 1469 1472 10.1016/S0140-6736(04)16108-4 15121412

[B15] Editorial Migration of health workers: an unmanaged crisis The Lancet 2005 365 9474 1825 16118902

[B16] Robert Putnam "Bowling Alone: The Collapse and Revival of American Community" (Simon and Schuster) 2000

